# The care of the patients with hipoplastic left heart syndrome in places of social and economic vulneability. An ethical analysis

**DOI:** 10.1590/0100-6991e-20233437-en

**Published:** 2023-03-31

**Authors:** ISAURA ELAINE GONÇALVES MOREIRA ROCHA, FLÁVIA LINS BEZERRA DE SOUZA FONSECA, JOSIMÁRIO SILVA

**Affiliations:** 1 - Universidade Federal de Pernambuco, Programa de Pós-graduação em Cirurgia - Recife - PE -Brasil; 2 - Universidade Federal do Cariri, Faculdade de Medicina - Barbalha - CE - Brasil

**Keywords:** Bioethics, Congenital Heart Diseases, Norwood Procedures, Palliative Care, Bioética, Cardiopatias Congênitas, Procedimentos de Norwood, Cuidados Paliativos

## Abstract

The birth of a child means hope and joy, particularly for the parents and the healthcare team. When this child is born with a severe malformation and a poor prognosis, as in the case of hypoplastic left heart syndrome, the scenario is one of great uncertainty and emotional suffering. The role of the health team becomes fundamental for the identification of conflicts of values and for the search for shared decisions that promote the best benefit to the child. When the diagnosis is made during fetal life, it is necessary to develop counseling strategies appropriate to the context of each family. In places with limited care resources, precarious prenatal care and short temporal conditions, the recommended counseling is compromised. Indication of treatment requires technical competence and a detailed analysis of ethical issues, and consultation with institutional clinical bioethics services or commissions is important. The article proposes to address the moral conflicts of two clinical cases and the respective bioethical analysis that involves principles and values in contexts of vulnerability and uncertainty, contrasting two situations where the indication of treatment was based on accessibility to treatment.

## RESUMO

O nascimento de uma criança significa esperança e alegria, particularmente para os pais e para a equipe de saúde. Quando essa criança nasce com uma malformação grave e de prognóstico reservado, como acontece na SHCE, o cenário é de grande incerteza e sofrimento emocional. O papel da equipe de saúde torna-se fundamental para a identificação de conflitos de valores e para a busca de decisões compartilhadas que promovam o melhor benefício à criança. Quando o diagnóstico é feito em vida fetal, é necessário a elaboração de estratégias de aconselhamento adequadas ao contexto de cada família. Em locais com recursos assistenciais limitados, acompanhamento prénatal precário e condição temporal curta, o aconselhamento preconizado fica comprometido. A indicação do tratamento requer competência técnica e uma análise pormenorizada de questões éticas, sendo importante a consultoria aos serviços ou comissões de bioética clínica institucional. O artigo propõe a abordar os conflitos morais de dois casos clínicos e a respectiva análise bioética que envolve princípios e valores em contextos de vulnerabilidade e incertezas, contrapondo duas situações onde a indicação do tratamento baseou-se na acessibilidade ao tratamento.

## INTRODUCTION

Hypoplastic left heart syndrome (HLHS) is possibly the most serious neonatal congenital heart disease, characterized by inadequate anatomical and functional development of the structures on the left side of the heart, with the inability of the left ventricle to perform systemic perfusion^1^. It has a varied anatomical spectrum, being a lethal pathology if not treated. Survival strategies have been offered over the last four decades and current treatment possibilities for the condition include the Norwood’s palliative surgery, neonatal heart transplantation, and compassionate or supportive care^2^ ^,^ ^3^.

Although surgical interventions are the treatment modalities that show the best results in terms of survival, allowing the modification of the natural history of the disease, the prognosis is uncertain in most cases, since even in adequate care conditions and with early interventions, there is a considerable risk of severe postoperative complications, neurological sequelae, and prolonged hospital stays, with physical and emotional suffering for the patient and family^4^ ^,^ ^5^. Thus, compassionate treatment is considered in many situations^6^.

In populations with limited health resources, counseling parents and the most viable therapeutic decision become a challenge, generating discussions about the most rational strategy to be followed. In the field of ethics, moral conflicts are established on decision-making, involving bioethical principles, values, and beliefs, requiring that teams, in addition to technical competence, have skills in deliberating on uncertain cases and avoiding reckless conducts, seeking a morally correct decision.

## CLINICAL CASES

### Case 01

Primiparous pregnant woman, suspected fetus with HLHS in the first trimester of pregnancy. Parents live in a medium-sized city in Northeast Brazil and belong to an economically favorable social class. After diagnostic confirmation in the second trimester, they were referred for prenatal counseling at a national reference service in the surgical treatment of HLHS. The delivery was scheduled with the necessary support. The male infant was born at term and underwent the first stage of Norwood palliative surgical correction in the first days of life. The parents previously signed the informed consent form about the risks of the proposed procedures. The child remained hospitalized for the first nine months of life, with his mother. The initial surgical results were considered successful, the child evolved without major clinical complications and showed good neuropsychic development for his age. At two years of age, the last stage of surgical correction was scheduled, which was performed by the same team. The child was operated on, but died in the postoperative period.

### Case 02

Primiparous woman with fetus diagnosed with HLHS in the second trimester. The parents are farmers, living in a poor community in the interior of Northeast Brazil. The couple was referred for prenatal counseling and follow-up at a tertiary service in the state capital, where they had access to scheduled delivery. The female baby was born at term, and the medical option was to indicate compassionate treatment. The child evolved with intense central cyanosis, low weight and mild delay in motor neuropsychic development. The family was instructed to maintain follow-up in a Cardiopediatrics service, but the follow-up did not happen satisfactorily due to the distance from her home and financial difficulties. Medical advice began to be given at the local health center, and parents, on their own initiative, began to perform semi-annual or annual echocardiograms. The child is currently four years old and has been proposed for evaluation for heart transplantation.

## CONTEXTUALIZATION

This study was approved by the Ethics Committee of Centro Universitário Unijuazeiro under protocol number 5.764.508. Informed consent was obtained from parents or legal guardians. The article presents two cases of HLHS that had the opportunity to be diagnosed in the prenatal phase, both residing in the interior of the Northeast of Brazil, but managed differently due to the issue of accessibility to treatment. In case 01, more educated parents with resources to seek advice and treatment in a specialized center opted for the Norwood’s palliative procedure. In case 02, parents had little education and resources, the child was born in a non-specialized service, and treatment was decided by the medical team. Surprisingly, the child survived with little or no medical intervention, beyond the expected average, reflecting in this case a more favorable anatomical spectrum of the malformation.

Although case 01 had a late death outcome, the parents demonstrated serenity and a sense of accomplishment after the mourning period, as they were able to offer their child a chance of survival. In case 02, compassionate treatment was decided due to a vulnerable social context, without adequate medical or psychological care support in subsequent years. However, parents on their own sought the echocardiography service at least once a year in the expectation of “some improvement” in the cyanotic and “inoperable” child.

The survival of needy children without any specialized intervention raises reflections on whether the treatment option was in fact the most reasonable from a bioethical point of view in the specific situation, given the advances in the treatment of this serious cardiac malformation in specialized centers worldwide, which has considerably increased these children’s life expectancy, allowing them to reach adulthood and become candidates for a heart transplant^7^ ^-^ ^9^.

The questions asked concern the accessibility to treatment within an unfavorable socioeconomic context. Should the State pay for all treatments for children who are diagnosed with HLHS? Is access to treatment at a reference center only for those who have the economic conditions to pay for it? If there is a surgical option validated in the medical literature that can modify the natural course of the disease and allow future survival, shouldn’t it be more widely used?

HLHS, like most congenital heart diseases, has a highly variable anatomical spectrum, the extreme cases directly influencing surgical results. In about 30% of patients, there are associated chromosomal anomalies, such as Turner syndrome, chromosomes 18 or 13 trisomy, translocations, and deletions, in addition to extracardiac anomalies, such as cystic hygroma, diaphragmatic hernia, encephalocele, omphalocele, facial teratoma, hydrocephalus, renal anomalies, and congenital clubfoot^10^ ^,^ ^11^. Faced with the various uncertainties and cases with unfavorable cardiac anatomy, wouldn’t surgical indication be a therapeutic futility, especially in poorer regions? If this hypothesis is likely, should it be funded with public resources?

Given that the Northeast Region has significant financial and geographic limitations, with few cardiopediatrics and pediatric cardiac surgery services, generally centered in state capitals, having few neonatal intensive care beds and serious failures in basic prenatal care, would the resources optimization to treat a complex heart disease with HLHS be justifiable, knowing that the main cause of neonatal death in the region is still prematurity^12^ ^,^ ^13^? Wouldn’t resources directed towards very complex pathologies with a reserved prognosis be taking away real chances of survival for children who manage to be born with less complex pathologies? How should the physician act from an ethical point of view when establishing this diagnosis in fetal life or, in the most common scenario, shortly after birth?

## BIOETHICAL DISCUSSION

Professionals who are dedicated to the care of fetuses and newborns with serious pathologies, which potentially can be treated with “aggressive and invasive” medical procedures, as occurs in HLHS, are often faced with complex decisions of high clinical and ethical uncertainty. Together with the parents, they have the responsibility to decide according to the child’s “best interest” criterion. However, there are differences of opinion about what is considered the best interest of the sick child, as there are different moral perceptions of decision makers^14^ ^,^ ^15^.

In situations where parents have low levels of education and precarious socioeconomic conditions, it is common for them to give little or no opinion and delegate the responsibility for directing the conduct to be adopted to the medical teams. Hence the difficulty of making the best decision in the context of ethical uncertainty in the search to identify the best interests of the child with a complex congenital heart disease.

Medicine, like clinical ethics, are practical disciplines based on the logic of probability rather than the logic of certainty. This means that, although in decision-making uncertainty must be reduced to the minimum possible, it cannot be totally eliminated. The desired objective would be not to reach certainty of a conduct, since this would not occur, but a balanced and reasonable decision possible within the different contexts.

Deliberation is an ethical-clinical method for choosing a prudent decision in difficult clinical cases where there are uncertainties as to the most appropriate course of action, and it is structured on three fundamental pillars: clinical facts (diagnosis, prognosis, and treatment); values such as the moral structure of human beings; and the duties that oblige physicians to promote actions that benefit the patient^16^ ^,^ ^17^. Although there are other decision-making methods within the scope of medical ethics, moral deliberation promotes greater clarity, less ambiguity, and even submits the decision taken to a consistency test, removing the idea that a good decision is based only on the physician’s experience.

No technical decision should be made without considering the moral values of the individual and, in this scenario, the deliberative process becomes an excellent tool to help clinical decision-making in neonatology of high ethical complexity, such as the management of a newborn with HLHS. The clinical evolution of a patient who has a life-threatening disease with a poor prognosis, which has many limitations and the need for extreme measures, needs to be deliberate in seeking to identify a prudent course of action that meets the patients’ real needs and in tune with the preservation of their dignity. In pediatrics, deliberating for decision-making becomes much more complex, but no less necessary, especially in diseases where the recommended treatment is controversial and in cases in which the outcome is predominantly unfavorable.

### Principles of Bioethics

The fundamental principles of biomedical ethics, elaborated in the 1970s in the United States of America, were the great beacons of bioethics in Brazil, in such a way that even today when we refer to bioethics, it is inevitable to think of autonomy, beneficence, non-maleficence, and justice^18^.

Although these principles are still widely used in decision-making, they should not be invoked in an uncritical or hierarchical way. Placing hierarchy in ethical principles can generate a dangerous risk of interpretation, as happens in situations where, for example, the principle of autonomy is considered the most important. Autonomy is a principle of great relevance that must be respected and considered, when the patient, with capacity and competence, is able to process the risks and possible benefits of an intervention from the available information, has time to process this information, and is able to self-determination, to accept or refuse an intervention proposal, regardless of whether it provides a clinical benefit or not.

In a condition of social, economic, or biological vulnerability, the principle of autonomy may not be the most important, or what best protects the patient ethically, leaving it to the physician to invoke the principles of non-maleficence or justice. Therefore, greater knowledge is needed about decision-making using bioethical principles. And going beyond principles, decision-making needs to include values, and the use of moral deliberation becomes an excellent tool. It is fundamental to understand that bioethical principles are values, but the opposite is not always true, and in the doctor-patient relationship, there are more values than principles to be preserved.

When decisions are taken based on principles of bioethics, with regard to advanced life support limitations, one should consider the principle of non-maleficence, which is characterized by medical conduct that aims not to institute or suspend an intervention that does not promote benefit to the patient. Doctors must use all possible resources, as long as they have the patient’s good as their objective, but when it is not achieved, it is ethical that it be replaced or withdrawn. It is the doctor’s duty not to abandon the patient when the prognosis is very limited. On the principle of beneficence, the physician must use the best scientific evidence to benefit the patient. It is an obligation to take action, to carry out medical interventions to treat the patient. It is ensuring essential care for the patient. From the perspective of the principle of justice, one should think about the efficient use of available resources^19^ ^,^ ^20^ ^,^ ^22^. When the available resources fail to effectively promote a benefit to the patient, it is ethically appropriate not to implement or suspend it, but without failing to promote strict control of the symptoms that may cause suffering. It is ethically correct to institute a treatment when it is not clearly ineffective, but if the treatment does not result in the patient’s good, it should not continue, as insistence on it would be an imprudent action. Obtaining informed consent as a guarantee of the decision-making process and the autonomy of the patient or his legal representative becomes fundamental in this process.

In the tradition of medicine built over time, it is considered that if a doctor has certain technological means, she/he must use it, always aiming at improving the patient’s clinical condition or illness. In certain situations, however the limits between a real benefit and a foreseeable damage are very tenuous, promoting poor quality patient survival, with invasive and painful interventions, and low expectations of alleviating the patient’s suffering or reversing the disease. This attitude of overvaluing health technology is known as the technological imperative and this logic often violates fundamental principles of human dignity, such as patient autonomy, right of refusal, intimacy, and other values^21^.

Defining a previous care plan that is tailored to the patient’s needs is what is expected of a team, and if there are moral conflicts that make decision-making difficult, an opinion from the Clinical Bioethics Commission is necessary.

### Parenting advice

HLHS is one of the most frequently diagnosed intrauterine cardiac anomalies and its recognition in routine ultrasound examinations does not bring major challenges to the operator, being possible from the 14^th^ week of gestation. Fetal echocardiography establishes anatomical details with great precision, evaluates the evolution of lesions and possible hemodynamic conditions at birth. With this data, parents should be referred for counseling with a specialized team, who have the duty to inform them about treatment alternatives, risks, and benefits, as well as likely scenarios if the child remains untreated. This informative process needs to be gradual and progressive, with empathy and with the necessary support for the pregnant woman and her family, and should be carried out impartially, avoiding following recommendations that the doctor or team believe to be “the most reasonable treatment option”, or the one that is usually practiced in that service^5^ ^,^ ^25^ .

The informed consent process is a legal and routinely recommended instrument in several services after diagnostic confirmation. However, it is not standardized as to the bioethical balance concerning the extent and limits of its application, varying as practiced by the obstetrician, neonatologist, pediatric cardiologist, and cardiac surgeon, with regard to termination of pregnancy, surgical treatment or supportive care^6^ ^,^ ^22^.

There must be clarity in the terms of the informed consent form if the surgical option has been chosen. The team has the responsibility not only to support the decision taken, but also to inform the parents about the best institutions to follow up on the patient’s treatment. The family’s process of understanding and autonomy need to be ensured, considering the uncertainties about the child’s future, as well as about the various stages of treatment.

### HLHS challenges in Brazil - from diagnosis to treament

In regions with greater social vulnerability in Brazil and in some Latin American countries, the intrauterine diagnosis of HLHS is not frequently performed, usually being late or after birth. This factor constitutes an important limiting factor in the newborn’s immediate management. When a child with this pathology is born in peripheral and unprepared units, the prognosis is aggravated by the lack of infrastructure and qualified professionals, added to the difficulty of transporting a neonate in critical condition to reference centers far from the place of birth. It is also a bioethical conflict for the professionals involved in this initial care and its influence in making the “best possible decision” in the face of unfavorable contexts.

Some parents are able and willing to dedicate themselves fully to their child’s care, using all available approaches and resources. This option cannot be suppressed based on supposedly more adequate and rational criteria such as the family’s socioeconomic status, or this would contradict the bioethical principle of justice^23^ ^,^ ^28^. The principle of autonomy of the parents must also be respected, after making sure that there was clarity in the conveyed information. The objective, therefore, is to build a path to create possibilities for choices, always making it clear when counseling parents that raising a child with this anomaly requires full dedication from the family. Several hospitalizations will be required during treatment, associated with emotional exhaustion and financial burden due to the chosen therapy.

Brazil has reference centers with almost 30 years of experience and successful reports of the Norwood surgery and increasing advances in hybrid procedures and postoperative care, with results comparable to the best centers in the world^29^ ^-^ ^32^. Nonetheless, the qualified services are mainly concentrated in the Southeast and South regions, being of difficult access for most cases from other regions of the country. There is a need for greater training and better preparation of professionals and health services involved in the care of these children and pregnant women, with greater awareness on the subject. Likewise, financial resources should be optimized for the creation of decentralized services, so that the access of the needy population is facilitated. While this favorable scenario does not become a reality, it is necessary to seek a bioethical balance, with respect to the fundamental principles of human beings and to life, where the professional is not simply failing to offer a chance for a surgical approach to heart disease, but showing how this choice is possible given the reality and existing results.

## CONCLUSION

Patients with HLHS usually have a poor prognosis and high mortality, even with all the advances in surgical techniques employed and advanced life support. The decision-making regarding surgical procedures should not be based only on the possibility of promoting improvements in the patient’s functional condition for a certain period of time, but also on future conditions regarding quality of life, and should involve a diligent and detailed assessment of the patient’s clinical conditions and limitations, an analysis of possibilities according to available resources and understanding of the disease.

As this is a clinical condition with many limitations in terms of prognosis and quality of life, it is necessary to deliberate on the clinical facts, values, and duties, as it is up to the professional to identify what would be a responsible, prudent, and achievable course of action. By deliberating, we are moving away from the dilemmatic view that reduces decisions to just two totally opposite possibilities, which fails to protect a greater number of values involved in the clinical case.

In a complex case, such as HLHS with great uncertainty as to the results, a careful assessment of the real indication for a surgical intervention is necessary, so that it is not just a surgical adventure. In cases of patients with little functional reserve, poor prognosis, and where there are marked uncertainties as to the result that promotes an adequate quality of life, the promotion of palliative care becomes desirable approach, avoiding futile procedures, which can be offered in outpatient and home follow-up, and in situations of aggravations more significant or depending on interventions, be carried out in the hospital environment.

Effective, progressive, and clear communication promotes greater acceptance for parents in the face of the uncertainties of each case, and allowing time to emotionally manage the whole situation.
